# Long-Term Prognosis among COVID-19 Patients: The Predictive Role Played by Hyperinflammation and Arrhythmic Disorders in Fatal Outcome

**DOI:** 10.3390/jcm12175691

**Published:** 2023-08-31

**Authors:** Domenico Cozzolino, Ciro Romano, Celestino Sardu, Riccardo Nevola, Giuseppina Rosaria Umano, Luca Rinaldi, Luigi Elio Adinolfi, Christian Catalini, Aldo Marrone, Maurizio Municinò, Ferdinando Carlo Sasso, Raffaele Marfella

**Affiliations:** 1 Department of Advanced Medical and Surgical Sciences, University of Campania L. Vanvitelli, 80138 Naples, Italy; domenico.cozzolino@unicampania.it (D.C.); ciro.romano@unicampania.it (C.R.); celestino.sardu@unicampania.it (C.S.); luca.rinaldi@unicampania.it (L.R.); luigielio.adinolfi@unicampania.it (L.E.A.); c.catalini@libero.it (C.C.); aldo.marrone@unicampania.it (A.M.); ferdinandocarlo.sasso@unicampania.it (F.C.S.); raffaele.marfella@unicampania.it (R.M.); 2 Department of the Woman and the Child and of General and Specialized Surgery, University of Campania L. Vanvitelli, 80138 Naples, Italy; giuseppinarosaria.umano@unicampania.it; 3 Department of Forensic, Evaluative and Necroscopic Medicine, ASL Napoli 2 Nord, 80027 Naples, Italy; municinodrmaurizio@gmail.com

**Keywords:** COVID-19, long-term follow-up, all-cause mortality, death predictors

## Abstract

Limited data are available on outcomes among COVID-19 patients beyond the acute phase of the disease. All-cause mortality among our COVID-19 patients one year after hospital discharge and factors/conditions associated with death were evaluated. All patients discharged from our COVID center were periodically evaluated by clinical assessment and by digital healthcare registry consultation. All findings acquired on discharge day represented the baseline data and were utilized for statistics. Of the 208 patients admitted, 187 patients were discharged. Among these, 17 patients died within 12 months (non-survivors). Compared to survivors, non-survivor patients were significantly (*p* < 0.05) older, exhibited significantly greater comorbidities and prevalence of active malignancy, heart failure, and arrhythmias, and showed significantly higher circulating levels of B-type natriuretic peptide, troponin, C-reactive protein, and d-dimer, as well as a longer heart-rate-corrected QT interval and significantly lower values for the glomerular filtration rate. Following multivariate analysis, cancer, arrhythmias, and high C-reactive protein levels were found to be factors independently associated with death. At the one-year follow-up, about 9% of patients discharged from our COVID center had a fatal outcome. Ageing, myocardial injury, impaired renal function, and, in particular, cancer, hyperinflammation, and arrhythmias represented strong predictors of the worst long-term outcome among COVID-19 patients.

## 1. Background

Severe acute respiratory syndrome coronavirus 2 (SARS-CoV-2) infection, responsible for coronavirus disease 2019 (COVID-19), firstly reported in China at the end of 2019, is characterized by high morbidity and mortality. Fortunately, the current trend in the prevalence of new coronavirus cases and deaths is a worldwide decrease. As of 21 May 2023, the WHO globally reported over 766 million confirmed cases and over 6.9 million deaths, secondary to acute respiratory distress syndrome and/or single or multiple involvement of other organs/systems [1].

Adverse cardiovascular events, including arterial thrombosis, venous thromboembolism, acute coronary syndrome, stroke, myocarditis, pericarditis, and arrhythmias, have been recognized as the most common complications among COVID-19 patients during the acute phase of the disease [2,3]. Moreover, both right and left systolic and diastolic ventricular dysfunction have been identified as common cardiovascular complications during SARS-CoV-2 infection [4,5,6]. Arrhythmias and their associated abnormalities in the local propagation of electrical impulses between myocytes seem to play a negative prognostic role in in-hospital outcomes among COVID-19 patients [7,8,9]. However, given that COVID-19 is a multisystemic disease, as well as the expected lung damage and the cardiovascular system involvement, it does not spare other organs/systems, including the kidneys, the liver, the brain, the peripheral and central nervous systems, and the immune system [10,11,12].

To date, while the current literature contains an abundance of data on so-called long COVID, understood as a long-lasting condition secondary to SARS-CoV-2-related involvement of some organs and systems, especially the immune system, the lungs, and the cardiovascular system, the data on outcomes and on final causes of death among COVID-19 patients beyond the acute phase of the disease are limited. However, one of the questions often debated among physicians is whether or not injury of the heart and/or a condition of systemic hyperinflammation, both of which are expected secondary infections to viral infection, are implicated in life expectancy among COVID-19 patients who survive the critical phase of the illness [13]. In this regard, although some blood purification strategies that aim to remove inflammatory mediators have been evaluated in COVID-19 patients, available data are not conclusive [14,15].

However, as reported by a recent study which mainly focused on cardiovascular complications among patients with COVID-19, adverse events are mostly confined to a time range of 1 to 12 months after hospital discharge [16]. In an analogous study, we followed over a period of 1 year all the patients consecutively admitted to our COVID center and discharged soon after normalization of their clinical status. The present study aimed to evaluate all-cause mortality at the one-year follow-up among a cohort of COVID-19 patients discharged from a dedicated COVID center in Campania, a region of southern Italy, and to explore some of the factors potentially predictive of a poor prognosis in such patients.

## 2. Materials and Methods

This was an investigative, monocentric, and prospective study conducted at the COVID Center of the University of Campania L. Vanvitelli, Naples, Italy. The study was conducted in accordance with the Declaration of Helsinki and approved by the Ethics Committee of the University of Campania L. Vanvitelli (Approval Code: 10117/2020). The cohort of patients consisted of all consecutive subjects (n. 208; M/F: 128/80) admitted between 15 December 2020 and 15 May 2021. Each patient had received a diagnosis of COVID-19 on the basis of a real-time reverse transcriptase-polymerase chain reaction for SARS-CoV-2. All patients were closely monitored through the entire hospitalization period. Patient histories were accurately collected at admission, whereas physical examinations and laboratory and instrumental investigations were performed both at hospital admission and at discharge. All patients underwent standard laboratory assays, standard 12-lead electrocardiogram (ECG), lung imaging examinations, including lung computed tomography score (LCTS) and lung ultrasound score (LUS), and bedside echocardiography. Acute myocardial infarction, stroke, arterial/venous thrombosis/embolism, serious life-threatening arrhythmias, pulmonary embolism, and decompensated heart failure were considered as major cardiovascular events. 

The ECG trace was continuously monitored for all patients during the entire period of hospitalization by means of a telemetric system (Dräger Vista 120 S, Drägerwerk AG, Lübeck, Germany), in order to monitor and record chronic arrhythmias and/or eventual new-onset arrhythmic episodes, respectively [7].

Laboratory measurements were carried out inside our institution using available commercial kits. 

Briefly, a 12-lead ECG was recorded at a standard speed of 25 mm/s both on admission day and at discharge. Particular attention was paid to accurately evaluation of the QT interval, which was measured from the beginning of the QRS complex to the end of the T wave. The point at which the T wave returns to baseline was defined as its end. In patients with bundle branch block, the QT interval was measured according to the Bogossian formula [17]. The data were excluded from the study if the T wave was not reliably measurable. QT dispersion was defined as the difference between the maximum and the minimum QT intervals for any of the 12 leads on the ECG trace in each patient. The Fridericia formula was utilized to calculate the heart-rate-corrected QT interval (QTc); QTc = QT/cube root of the RR interval [18].

The LCTS is a semi-quantitative method utilized to describe the degree of impairment in each of the five lung lobes, with a total score ranging from 0 (no involvement) to 25 (maximum involvement) [19]. The LUS is commonly acquired on the standard sequence of 14 peculiar anatomic landmarks, with a score ranging between 0 and 3, and is based on the involvement shown in the ultrasound image; the lung total score was calculated by adding all the scores together and ranged from 0 (no involvement) to 42 (maximum involvement) [20].

Echocardiographic scans were performed to mainly evaluate the regional and global systolic function of the left ventricle (LVEF), according to standard guidelines [21], with a complete examination being performed when necessary.

The findings acquired on the day of discharge from our COVID center for each patient taking part in the present study were defined as baseline data and were utilized for statistical analyses. Each of our patients was evaluated every 3 months until 12 months after discharge in order to assess functional health status by means of the Barthel scale. In addition, SANI.A.R.P. Campania was systematically consulted in order to inquire about every eventual change in our patients’ state of health during the intervals between the quarterly consultations. The SANI.A.R.P. Campania is a digital healthcare registry able to provide statistical, economic, and clinical–epidemiological data of all citizens of Campania. This system allows accredited healthcare personnel to access the patients’ electronic health records, which provide for each resident complete data on pharmacological and specialist services and an extensive collection of clinical health documentation issued by public and private health authorities. 

### Statistical Analysis

Continuous variables distribution was investigated for normality with the Kolmogorov–Smirnov test. Continuous variables were expressed as the median and the interquartile range (IQR) and categorical variables were expressed as frequencies. Differences for continuous variables were investigated with Student’s *t*-test for independent samples and the Mann–Whitney U test as appropriate. Chi-square tests were performed for differences in categorical variables. Post hoc analysis with the Bonferroni correction for multiple comparisons was performed when appropriate. Variables which demonstrated association (*p* < 0.05) with death after comparison of the characteristics of survivors were included in the univariate analyses. Logistic transformation was performed for those variables not displaying a normal distribution to accomplish normality in logistic regression analyses. Univariate logistic regression analyses were performed to identify candidate variables for multivariate analysis to estimate the risk of presenting death. Statistically significant variables in the univariate analysis were included in the multivariate analysis. All the analyses were performed using SAS^®^ University Edition (SAS Institute Inc., Cary, NC, USA). *p* values < 0.05 were considered statistically significant.

## 3. Results

The mean age of our cohort of COVID-19 patients (n. 208; 128 M/80 F) admitted to our COVID center was 70.1 years (range: 26–94 years). Among our 208 patients, 52 subjects (25%) exhibited diabetes mellitus, 53 (25.4%) obesity, 27 (13%) hyperlipidemia, 24 (11.5%) chronic liver disease, 22 (10.6%) chronic kidney disease, and 35 (16.8%) chronic obstructive pulmonary disease, while 94 (45%) were a current user of tobacco, 16 (7.7%) were suffering from auto-immune diseases, and 8 (3.8%) were suffering from active malignancy. Finally, 4 (1.9%) had previously received organ transplantation, 10 (4.8%) were affected by rare diseases, 118 (56.7%) by arterial hypertension, 30 (14.4%) by coronary artery disease, and 13 (6.3%) by heart failure, while 46 (22.1%) had previously received a diagnosis of arrhythmias and 10 (4.8%) had previously received an anti-arrhythmic device implantation. Globally, the average number of comorbid conditions was 2.8. No patient was found to have been vaccinated for SARS-CoV-2 infection at the time of hospital admission. During hospitalization for COVID-19, 152 patients (73.1%) developed acute respiratory distress syndrome (ARDS). Moreover, 75 patients (36%) underwent continuous positive airway pressure (CPAP) and/or non-invasive ventilation (NIV), and 10 (4.8%) underwent orotracheal intubation (OTI) and invasive ventilation. The timing and modalities of invasive and non-invasive ventilation have been described in a previous study of our group [22]. A total of 21 patients (15 M/6 F) died during the in-hospital period.

### 3.1. At Discharge (Baseline)

All patients discharged from our COVID-19 center soon after normalization of their clinical picture (187 patients; 113 M/74 F) entered the follow-up program. The median age of these patients was 73 years (range: 26–94 years), and the average number of comorbid conditions was 2.5. Tobacco was currently used by 83 (44.4%) of the patients. The results of consideration of the main comorbid conditions among our patients were that 42 (22.5%) were obese and 42 (22.5%) were affected by diabetes mellitus, while 8 (4.3%) were suffering from active malignancy, 98 (52.4%) from arterial hypertension, 8 (4.3%) from heart failure, and 24 (12.8%) from coronary artery disease (Table 1).

#### 3.1.1. Cardiac Telemetric Monitoring

Among 187 patients discharged, 60 (32.1%) of them had showed ECG traces consistent with rhythm disturbances recorded on cardiac telemetry (Table 1). Of these, 33 (55%) patients had developed new-onset arrhythmias, whereas the remaining 27 (45%) were affected by a chronic condition of arrhythmia.

#### 3.1.2. Laboratory Assays

Laboratory measurements of the main blood parameters are depicted in Table 1. Values for the glomerular filtration rate (GFR) and hemoglobin concentrations were lower than the normal range in 30 (16.0%) and 41 (21.9%) of our COVID-19 patients, respectively, whereas those for circulating B-type natriuretic peptide (BNP), troponin, C-reactive protein (CRP), ferritin, interleukin-6, and d-dimer were higher than the normal range in 16 (8.6%), 11 (5.9%), 97 (51.9%), 97 (51.9%), 181 (96.8%), and 94 (50.3%) patients, respectively. The median values for the main laboratory parameters assessed in our cohort of patients and the reference ranges of such parameters, as reported by commercial kits commonly used in our institution, are shown in Table 1.

#### 3.1.3. Lung and Cardiac Instrumental Investigations

All patients admitted to our COVID-19 center showed a manifest involvement of the lungs. Average values for the LCTS and the LUS are reported in Table 1. Left ventricle systolic performance was shown to be particularly impaired (LVEF < 45%) in 12 patients (6.4%). Average values for the LVEF are depicted in Table 1. 

### 3.2. At One-Year Follow-Up

After a one-year follow-up, a total of 17 (9.1%) deaths were adjudicated among our 187 COVID-19 patients discharged from hospital (non-survivor group). Among non-survivor patients, only two of them received vaccination for SARS-CoV-2 after discharge. On the basis of data acquired on discharge day (baseline findings), survivor patients were significantly (*p*: 0.0001) younger and exhibited a significantly (*p*: 0.003) lower number of comorbidities when compared to non-survivor patients (Table 2). Moreover, prevalence of active cancer, heart failure, and arrhythmias among survivors was significantly (*p* < 0.05, at least) lower than in patients who died (Table 2). 

Circulating levels of BNP, troponin, CRP, and d-dimer among non-survivor patients were found to be significantly (*p* < 0.02, at least) higher than those among survivors (Table 2). Values for the glomerular filtration rate among non-survivors were significantly (*p*: 0.0003) lower when compared to those in survivor subjects, whereas hemoglobin concentrations were found to be only slightly lower (Table 2). Cardiac and pulmonary instrumental findings showed no significant differences when the two cohorts were compared, except for QTc, which was significantly longer among non-survivor patients as compared to survivors (Table 2).

The univariate analysis showed that the factors/conditions associated with the risk of death in our cohort of COVID-19 patients were advanced age, active cancer, heart failure, arrhythmias, lower values for the glomerular filtration rate, and higher circulating levels of BNP, troponin, CRP, and d-dimer (Table 3). The multivariate analysis showed that the factors independently associated with the risk of death were active malignancy, rhythm disorders, and abnormal values of CRP, whereas an independent association of advanced age with the risk of death was only slight (Table 4).

Among non-survivor patients, ten subjects died within 3 months of discharge, four subjects died between 4 and 6 months, three subjects died between 7 and 9 months, whereas no subjects died between 10 and 12 months of discharge (Table 5). Serious cardiovascular accidents accounted for 42.2% (7 patients) of all adjudicated deaths at a follow-up of 12 months, active cancer for 35.3% (6 patients), and other causes for the remaining 23.5% (4 patients) (Table 5).

## 4. Discussion

The present study has been conceived as a monocentric prospective evaluation of patients consecutively hospitalized with COVID-19 at our center. In contrast with a previous retrospective multicentric study which reported a 1-year mortality rate of about 18% among COVID-19 patients [2], the present study found an all-cause mortality rate of about 9%. Patients in this study mostly died within 30 to 50 days from discharge, and these findings are aligned with those reported by other authors who found a clustering of adverse prognoses within 1 month from discharge [2,16,23].

When compared to survivor patients, non-survivors in the present study were older and exhibited a larger clustering of comorbid conditions. The prevalence of active cancer and cardiac involvement—the latter being evidenced by a concomitant heart failure condition and/or chronic or new-onset arrhythmias, on the one hand, and by higher levels of BNP and troponin together with an elongated QTc interval, on the other hand—was greater among non-survivor patients. In addition, renal function was found to be somewhat impaired, as expressed by lower values for GFR, among the deceased patients of the present study. Furthermore, plasma levels of CRP and d-dimer, known as indices of hyperinflammation and of an unbalanced coagulative milieu, respectively, were significantly increased among non-survivor patients.

Following univariate analysis, the risk of all-cause death within 12 months after hospital discharge among our patients was found to be associated with older age, active cancer, some chronic and new-onset cardiovascular diseases, including heart failure and arrhythmias, and kidney failure. Moreover, the risk of death among patients in the present study was shown to be associated with elevated values of BNP and troponin, both markers of myocardial damage. In addition, there was evidence of an association of higher circulating levels of CRP and d-dimer, as the expression of a combined condition of infectious/hyperinflammatory status together with a coagulative imbalance, with the risk of all-cause death among our patients. Following multivariate analysis, augmented circulating levels of CRP, chronic and new-onset rhythm disturbances, and active malignancy were found to create an independent potential risk for all-cause death among patients of the present study at 1 year after hospital discharge, whereas older age was shown to be only slightly associated with death.

CRP is recognized as one of the main markers of systemic inflammation and/or severe infection, as a low-cost assay, as easily assessable, and, finally, as largely available. It is noteworthy that the findings of elevated CRP plasma levels and their strong association with a higher risk of all-cause mortality among non-survivors of the present study are in agreement with the results of previous works, which showed a strong association of a condition of hyperinflammatory status and/or a manifest myocardial injury related to SARS-CoV-2 infection with both short- and long-term adverse outcomes among COVID-19 patients [24,25,26]. In addition, CRP levels have been reported to be strongly correlated with the magnitude of myocardial injury in COVID-19 patients [3,27,28].

According to a recent work focusing on the role played by arrhythmias on adverse in-hospital outcomes in COVID-19 patients and similar other reports [7,9,29], the findings of the present study have confirmed that rhythm disorders confer a worse long-term outcome among COVID-19 patients, with a long-term risk about sixfold higher for all-cause mortality when compared to patients free of arrhythmias. Indeed, even though serious arrhythmia was fatal in solely two subjects (11.7%), about 71% of the patients who died exhibited rhythm disturbances at the one-year follow-up point. By analogy, a recent follow-up study conducted in a cohort of about 3600 COVID-19 patients found an about fourfold higher incidence in rhythm disturbance together with an equally higher incidence in all-cause mortality when compared to about 850 control subjects [2]. It is possible to hypothesize that arrhythmia is likely to represent a negative prognostic factor per se, irrespective of final cause of death. Furthermore, there is evidence that both the type and the time of onset of arrhythmias did not influence all-cause mortality [7]. Basically, the mechanisms potentially leading to arrhythmias in patients with SARS-CoV-2 infection are numerous. Firstly, it is possible to speculate that a direct SARS-CoV-2 injury to myocytes associated with a parallel virus-related condition of systemic hyperinflammation, both responsible for local electrical imbalance, could be a driving force leading to rhythm disorders [3,28]. Moreover, hypoxia due to lung disease and/or pulmonary embolism, as well as intravascular volume and electrolyte disequilibrium secondary to direct gastrointestinal and kidney virus-related derangement, could represent other factors implicated in the genesis of arrhythmia in COVID-19 patients. In addition, a prolongation of the QT interval, as a consequence of the direct or indirect action of some drugs on myocardial cells (for example, chloroquine and hydroxychloroquine, both often used in such patients during the acute phase of the disease) and electrolyte imbalance due to use of corticosteroid and/or diuretic drugs, has been demonstrated to create the potential for arrhythmic events in patients affected by COVID-19 [30]. It is noteworthy that a prolonged QT duration represents a factor often associated with severe arrhythmias potentially leading to death [31]. Interestingly, non-survivor patients in the present study showed significantly higher values for QTc and slightly higher values for QTc dispersion at discharge.

As expected, active cancer has been found to be an independent risk factor for death among patients in the present study. In fact, active malignancy accounted for about 35% of out-of-hospital mortality among our COVID-19 patients.

Surprisingly, the degree of lung involvement was not significantly different when the two cohorts of patients in the present study were compared. Indeed, respiratory failure was fatal in just one patient, who died due to acute respiratory distress. This evidence suggests that lung damage related to SARS-CoV-2 infection did not influence the long-term prognosis in our patients. Nobody among our patients was treated with anti-SARS-CoV-2 monoclonal antibodies, which have been found to somewhat protect against adverse short-term outcomes in COVID-19 patients [32]. Whether these treatment strategies in COVID-19 patients have a good efficacy/safety ratio or not in the long term remains an intriguing question.

### Study Limitations

The relatively small sample size represents an important limitation of the present study. Moreover, given that our ward is a dedicated COVID center, the present study lacked a control group, i.e., COVID-19-free patients, and this obviously precludes the possibility of robustly comparing outcomes between matched patients with and without COVID-19 and, therefore, of strongly evaluating the intrinsic role of SARS-CoV-2 in long-term prognosis. Furthermore, despite efforts to precisely collect patients’ personal histories, the possibility that previous arrhythmic episodes and/or other minor comorbid events (i.e., those occurred before hospitalization) have been underreported is not excluded. Finally, the causes of death were adjudicated in some cases in a physical hospital, but in other cases at the homes of patients by a generalist doctor, with the consequent limitations on precision of diagnosis.

However, homogeneity of data and low inter-observer variability due to standardization of patient observation and personal history collection might have minimized some of the methodological biases reported above. Indeed, unlike the majority of reports from the current literature of studies focused on COVID-19, which were mostly planned as multicenter studies, the present study was conceived as a unicentric study, with the advantage that laboratory measurements, instrumental diagnostic protocols, and data collection were rigorously standardized, so avoiding a number of confounding factors related to differences in patient general management protocols between participant centers, and therefore warranting the robustness of our findings.

Anti-SARS-CoV-2 vaccination could represent a potential confounding factor. Although the issue is controversial, vaccines have been linked by some authors to numerous adverse events, including myocarditis and ischemic stroke [33,34]. SARS-CoV-2 mRNA vaccines contain nucleoside-modified synthetic mRNA, encoding the viral spike protein. The affinity between the SARS-CoV-2 spike protein and cardiac and other cell membrane proteins could be responsible for an auto-immune reaction that results in hyperinflammation and injury to the endothelium and cardiomyocytes in susceptible individuals [35]. However, we believe that, in our study cohort, vaccination could not have adversely affected the results. In fact, no patient had been vaccinated before enrollment, and only two “non-survivor” patients received vaccination during follow-up.

## 5. Conclusions

A total of 17 patients (about 9%) affected by COVID-19 died within 12 months from hospital discharge. Severity of lung involvement did not influence outcome in this group of patients. Ageing, myocardial injury, cancer, inflammatory/coagulative imbalance and impairment in renal function were shown to be associated with a poor long-term prognosis in these patients. Although our results need to be treated with caution due to study limitations, arrhythmias and hyperinflammation seem to predict a fatal outcome among patients previously affected by COVID-19. If these findings are confirmed by larger and longer follow-up studies, a better understanding of the mechanisms involved could be provided, and a more adequate selection of patients deserving a closer follow-up could be available, helping to identify a more appropriate individual management strategy and, as a consequence, to mitigate the impact of the illness later in life.

## Figures and Tables

**Table 1:** Baseline demographic and clinical characteristics of patients recruited in the follow-up.

	All Patients (n 187)	Reference Range/Values
General features		
Sex (M/F)	113/74	-
Age (years)	73 (68–84)	-
Current tobacco user n (%)	83 (44.4)	-
Obesity n (%)	42 (22.5)	-
Diabetes mellitus n (%)	42 (22.5)	-
Chronic liver disease n (%)	20 (10.7)	-
Chronic kidney disease n (%)	15 (8.0)	-
COPD n (%)	26 (13.9)	-
Hyperlipidemia n (%)	23 (12.3)	-
Auto-immune disease n (%)	14 (7.5)	-
Active malignancy n (%)	8 (4.3)	-
Prior organ transplantation n (%)	2 (1.1)	-
Rare disease n (%)	8 (4.3)	-
Arterial hypertension n (%)	98 (52.4)	-
Heart failure n (%)	8 (4.3)	-
Coronary artery disease n (%)	24 (12.8)	-
Arrhythmia n (%)	60 (32.1)	-
ICD/PPM n (%)	7 (3.7)	-
Comorbidities	4 (3–4)	-
Laboratory findings		
Sodium (mmol/L)	137 (135–140)	135–146
Potassium (mmol/L)	4.2 (3.8–4.6)	3.5–5.3
Magnesium (mg/dL)	2.0 (1.8–2.2)	1.6–2.6
Calcium (mg/dL)	8.7 (8.4–9.0)	8.6–10.2
Creatinine (mg/dL)	0.83 (0.74–1.12)	0.51–0.95
GFR (mL/min/1.73 m^2^)	58.0 (49.0–82.0)	≥60
B-type natriuretic peptide (pg/mL)	55.3 (8.0–217.5)	≤125
Troponin (ng/mL)	5.1 (2.5–24.4)	≤33
C-reactive protein (mg/dL)	4.15 (1.55–11.6)	≤5
Ferritin (ng/mL)	1018 (201–2052)	13–150
Interleukin-6 (pg/mL)	30 (16–107)	≤5
D-dimer (ng/mL)	1200 (545–2280)	≤500 *
Hemoglobin (g/dL)	12.3 (9.9–12.3)	13–15/12.5–14.5 (M/F)
Instrumental findings		
Left ventricle ejection fraction (%)	54 (50–55)	55–65
Lung computed tomography score	8.5 (6–12)	0
Lung ultrasound score	14 (7–23)	0
QT (ms)	361 (345–363)	340–430
QTc (ms)	421 (391–462)	340–440/340–460 (M/F)
QTc dispersion (ms)	72 (51–100)	≤80

Values are showed as median (interquartile range) or as n (%); COPD: chronic obstructive pulmonary disease; GFR: glomerular filtration rate; ICD/PPM: implantable cardioverter defibrillator/permanent pacemaker; QTc: heart-rate-corrected QT interval; *: 10 × number of years if age > 50 years.

**Table 2:** Baseline main characteristics of the patients according to group.

	Survivors (n 170)	Non-Survivors (n 17)	*p* Value
General features			
Sex (M, %)	58.0	47.1	0.44
Age (years)	61 (52–71)	73 (68–84)	0.0001
Current tobacco n (%)	75 (44.1)	8 (47.1)	0.80
Obesity n (%)	39 (22.9)	3 (17.6)	0.77
Diabetes mellitus n (%)	37 (21.8)	5 (29.4)	0.54
Active malignancy n (%)	4 (2.3)	4 (23.5)	0.0004
Arterial hypertension n (%)	86 (50.6)	12 (70.6)	0.14
Heart failure n (%)	6 (3.6)	3 (17.7)	0.04
Coronary artery disease n (%)	20 (11.8)	4 (23.5)	0.24
Arrhythmia n (%)	48 (28.2)	12 (70.6)	0.0008
Comorbidities	2 (1–3)	4 (3–4)	0.003
Laboratory findings			
Creatinine (mg/dL)	0.81 (0.72–0.93)	0.83 (0.74–1.12)	0.50
GFR (mL/min/1.73 m^2^)	92.0 (75.0–102.0)	58.0 (49.0–82.0)	0.0003
B-type natriuretic peptide (pg/mL)	18.0 (10.0–43.0)	59.5 (47.0–281.0)	0.001
Troponin (ng/mL)	3.0 (2.0–8.0)	15.0 (6.0–89.5)	0.0004
C-reactive protein (mg/dL)	0.66 (0.27–1.80)	6.90 (3.20–11.70)	<0.0001
Ferritin (ng/mL)	479 (224–746)	1018 (201–2052)	0.10
Interleukin-6 (pg/mL)	20.9 (8.5–39.0)	30.0 (16–107.0)	0.29
D-dimer (ng/mL)	610 (346–1090)	1200 (545–2280)	0.01
Hemoglobin (g/dL)	13.4 (12.1–14.3)	12.3 (9.9–13.8)	0.056
Instrumental findings			
Left ventricle ejection fraction (%)	55 (51–55)	53 (50–55)	0.29
Lung computed tomography score	9 (6–12)	8.5 (6.0–12.0)	0.87
Lung ultrasound score	12 (8–18)	14 (7–23)	0.50
QT (ms)	360 (320–405)	363 (351–365)	0.95
QTc (ms)	393 (380–415)	421 (393–461)	0.02
QTc dispersion (ms)	70 (50–83)	72 (51–100)	0.09
Medical treatments			
Proton-pump inhibitors n (%)	80 (47.1)	8 (47.1)	1.00
Antihypertensive drugs n (%)	86 (50.6)	12 (70.6)	0.14
Antihyperglycemic drugs n (%)	36 (21.2)	5 (29.4)	0.65
Statins n (%)	20 (11.8)	3 (17.6)	0.47
Class I, III anti-arrhythmic drugs n (%)	45 (26.5)	7 (41.2)	0.19
Antiplatelet drugs n (%)	35 (20.6)	5 (29.4)	0.61
Oral anticoagulant drugs n (%)	21 (12.4)	3 (17.6)	0.52
Loop diuretics n (%)	19 (11.2)	4 (23.5)	0.14
ARNI n (%)	5 (2.9)	2 (11.7)	0.75
Anti-inflammatory drugs n (%)	19 (11.2)	3 (17.6)	0.49
Inhaled bronchodilator drugs n (%)	23 (13.5)	3 (17.6)	0.59
Antibiotics n (%)	19 (11.2)	3 (17.6)	0.49
Respiratory clinical findings			
ARDS n (%)	117 (68.8)	14 (82.3)	0.24
Need for CPAP/NIV	58 (34.1)	5 (29.4)	0.69
Need for OTI and IMV (ICU admission)	0 (1)	1 (5.9)	0.09

Values are presented as median (interquartile range) or as frequencies (%); ARDS: acute respiratory distress syndrome; ARNI: angiotensin receptor-neprilysin inhibitors; CPAP: continuous positive airway pressure; GFR: glomerular filtration rate; ICU: intensive care unit; IMV: invasive mechanical ventilation; NIV: non-invasive ventilation; OTI: orotracheal intubation; QTc: heart-rate-corrected QT interval.

**Table 3:** Univariate analysis of risk factors potentially associated with death.

	Univariate Analysis		
	OR	CI	*p* Value
Age	1.10	1.04–1.14	0.0003
Active malignancy	17.2	4.1–72.5	0.0001
Heart failure	5.8	1.3–25.8	0.02
Arrhythmia	6.05	2.01–18.09	0.0013
Number of comorbidities	2.28	0.91–5.67	0.08
Glomerular filtration rate	0.28	0.12–0.67	0.004
B-type natriuretic peptide	1.95	1.30–2.92	0.001
Troponin	2.33	1.51–3.62	0.0002
C-reactive protein	3.13	1.89–5.17	<0.0001
D-dimer	2.11	1.17–3.80	<0.0001
QTc	21.0	<0.1–152	0.76

QTc: heart-rate-corrected QT interval.

**Table 4:** Multivariate analysis of risk factors independently associated with death.

	Multivariate Analysis		
	OR	CI	*p* Value
Age	1.14	1.00–1.30	0.051
Active malignancy	21.2	4.1–111.7	0.0003
Heart failure	3.6	0.6–20.9	0.15
Arrhythmia	6.1	1.8–20.9	0.004
Glomerular filtration rate	1.7	0.2–15.2	0.62
B-type natriuretic peptide	1.6	0.7–3.5	0.29
Troponin	1.9	0.8–4.9	0.16
C-reactive protein	3.3	1.6–6.7	0.0009
D-dimer	0.9	0.4–3.4	0.88

Hyperglycemia: plasma glucose at admission ≥ 140 mg/dL.

**Table 5:** Causes and timing of death among the non-survivor cohort of patients.

Within 3 Months from Hospital Discharge	
Patient #1	Acute respiratory distress
Patient #2	Cancer of colon
Patient #3	Inflammatory bowel disease
Patient #4	Bowel obstruction
Patient #5	Prostate cancer
Patient #6	Hemorrhagic stroke
Patient #7	Severe sepsis
Patient #8	Cholangiocarcinoma
Patient #9	Decompensated heart failure
Patient #10	Acute myocardial infarction
Between 4 and 6 months from hospital discharge	
Patient #11	Ischemic stroke
Patient #12	Cancer of lung
Patient #13	Hemmorrhagic stroke
Patient #14	Serious arrhythmia
Between 7 and 9 months from hospital discharge	
Patient #15	Cancer of colon
Patient #16	Serious arrhythmia
Patient #17	Serious arrhythmia
Between 10 and 12 months from hospital discharge	
None	

## Data Availability

The data presented in this study are available on request from the corresponding author.
